# Balanced crystalloids vs. 0.9% saline in pediatric diabetic ketoacidosis: a systematic review and meta-analysis of randomized controlled trials

**DOI:** 10.3389/fped.2026.1879829

**Published:** 2026-07-09

**Authors:** Zeid Alkhairi, Charlie Kajo, Abdolaziz A. Zadeh, Dawood Khaja, Mohammad Alqaryuti, Nada K. Alsaleh, Rataj Alibrahim, Fahad Almuhannadi, Suod Al Hammad, Fatima Alqanea, Abdullatif Alfehaid

**Affiliations:** 1 School of Medicine, Royal College of Surgeons in Ireland, Medical University of Bahrain, Busaiteen, Bahrain; 2 College of Medicine and Medical Sciences, Arabian Gulf University, Manama, Bahrain; 3 Head of the Pediatric Department, King Hamad University Hospital, Muharraq, Bahrain

**Keywords:** acute kidney injury, balanced crystalloids, diabetic ketoacidosis, normal saline, pediatric, plasma-lyte, ringer's lactate

## Abstract

**Background:**

Fluid resuscitation is a cornerstone in the management of pediatric diabetic ketoacidosis (DKA). While 0.9% normal saline (NS) remains the conventional first-line fluid, concerns regarding its high chloride content and potential to induce hyperchloremic metabolic acidosis have led to increasing interest in balanced crystalloids. However, evidence comparing both strategies in pediatric populations remains limited and inconsistent.

**Methods:**

Five databases were searched from inception to 27 April 2026. The primary outcomes were time to resolution of DKA and incidence of new-onset acute kidney injury (AKI). Secondary outcomes included hospital and PICU length of stay, electrolyte disturbances, and major complications. Random-effects models were used. Risk of bias was assessed using the Cochrane RoB 2 tool, and certainty of evidence was evaluated using GRADE.

**Results:**

Five RCTs involving 320 patients were included. A modest but statistically significant decrease in the time to DKA resolution (MD: −1.60 h, 95% CI: −3.07 to −0.13) was associated with balanced crystalloids. Current evidence is insufficient to determine the effect of fluid choice on new-onset AKI due to substantial statistical uncertainty (RR: 0.55, 95% CI: 0.17–1.82; *p* = 0.325). No significant differences were observed in the other secondary outcomes; however, balanced crystalloids were associated with a lower incidence of hypokalemia (RR: 0.66, 95% CI: 0.46–0.93) and hyperchloremia (RR: 0.40, 95% CI: 0.21–0.78).

**Conclusion:**

Modest benefits in biochemical outcomes were associated with balanced crystalloids. However, due to the small evidence base and low certainty of evidence, it remains unclear if these advantages translate into improvements in clinically important outcomes, particularly rare complications such as cerebral edema and need for mechanical ventilation or inotropic support. Current evidence remains insufficient to support a transition from NS to balanced crystalloids as the initial fluid in pediatric DKA. Further large, multicenter RCTs are needed to better determine the clinical role of balanced crystalloids in this setting.

**Systematic Review Registration:**

https://www.crd.york.ac.uk/PROSPERO/view/CRD420261387598, identifier CRD420261387598.

## 1 Introduction

Diabetic ketoacidosis (DKA) is an acute and potentially life-threatening complication of diabetes mellitus, particularly in the pediatric population. It is defined by the triad of findings consisting of hyperglycemia (>11 mmol/L), serum ketosis (ß-hydroxybutyrate ≥3 mmol/L), and acidosis measured in either pH (<7.3) or bicarbonate (<18 mmol/L) (1). In the setting of absolute or relative insulin deficiency, reduced insulin activity leads to increased lipolysis, ketogenesis, and impaired glucose utilization, resulting in ketonemia, metabolic acidosis, and hyperglycemia. Moreover, an accelerated catabolic state further contributes to hyperglycemia and hyperosmolality through increased glucose production in the liver and kidneys (1). These processes lead to large loss of volume and electrolyte imbalances, including dilutional hyponatremia, whole body potassium depletion, and hyperchloremia. If not adequately screened and managed, these imbalances can result in more severe sequelae such as renal tubular injury, brain injury, and increased mortality. To appropriately prevent these complications, effective management of pediatric diabetic ketoacidosis requires a structured approach. This approach consists of fluid replacement, correction of electrolyte imbalances, and insulin therapy, with fluid resuscitation forming a critical component of the treatment.

Given physiological differences compared to adults, fluid resuscitation in the paediatric population requires careful consideration of various factors such as variations in fluid distribution, increased susceptibility to rapid osmotic shifts, and higher risk of cerebral oedema. According to the Canadian Paediatric Society, these differences necessitate a modified treatment approach in paediatric patients, including cautious intravenous fluid administration and delayed initiation of insulin following fluid resuscitation (2).

Conventionally, isotonic fluids, such as 0.9% saline, have been used as the initial fluid of choice given their established use and availability. Despite this, concerns have been raised regarding its high chloride content and, thus, the potential effects on the acid-base balance. In contrast, some studies have suggested that balanced crystalloids, such as Ringer's lactate (RL), are a safer alternative as they may minimize hyperchloremic metabolic acidosis, while also reducing the risk of cerebral and renal injury (3–6). In current clinical practice, both normal saline (NS) and balanced crystalloids are utilized during the treatment of DKA (2). However, differences may exist in clinically relevant outcomes, including time taken for full resolution of DKA, hospital stay duration, electrolyte complications, and new onset acute kidney injury (AKI). In our study, we aim to evaluate these outcomes to better define the favorable fluid strategy in pediatric DKA.

Notwithstanding increasing interest in balanced crystalloids, uncertainty and variation persist in current evidence. Therefore, a systematic review and meta-analysis of randomized controlled trials (RCTs) is imperative to integrate existing evidence in order to evaluate the clinical benefits and/or potential disadvantages of both fluid strategies. The review aims to compare the efficacy and safety of balanced crystalloids vs. 0.9% saline for fluid resuscitation in children with DKA.

## 2 Methods

### 2.1 Study registration

This study protocol, with the registration number of CRD420261387598, was registered in PROSPERO prospectively. This systematic review and meta-analysis adhered to the Preferred Reporting Items for Systematic Reviews (PRISMA) and recommendations of the Cochrane Handbook for Systematic Reviews of Interventions (7, 8). The completed PRISMA checklist is provided in the Supplementary Appendix Table S1. The final review fully adhered to the registered protocol with no deviations.

### 2.2 Literature search and study selection

Using PubMed, Embase, Scopus, Web of Science, and the Cochrane Library, a thorough search was carried out from database inception to 27 April 2026. Medical Subject Headings (MeSH) terms and free text keywords related to balanced crystalloids, 0.9% saline, and diabetic ketoacidosis were combined in the search strategy (Supplementary Table S2). No restrictions were applied regarding language or publication date.

All retrieved records were imported into Covidence for duplicate removal and screening. Two independent reviewers conducted a two-stage screening process, consisting of title and abstract screening followed by full-text assessment for eligibility. Any disagreements were resolved through discussion or consultation with a third reviewer. A PRISMA flow diagram was used to summarize the study selection process.

### 2.3 Eligibility criteria

Based on the PICO framework, studies were included if they satisfied the following requirements. The population (P) comprised pediatric patients (under 18 years of age) diagnosed with diabetic ketoacidosis using established criteria, including hyperglycemia, metabolic acidosis, low bicarbonate, and ketonaemia. The intervention (I) used balanced crystalloid solutions like Hartmann's solution, Ringer's lactate, and Plasma-Lyte (PL); meanwhile, the comparator (C) was 0.9% normal saline solution. The primary outcomes (O) were time to resolution of DKA (using set parameters, e.g., normalization of pH, bicarbonate, or ketones) and the incidence of acute kidney injury. While individual trials were largely powered around DKA resolution, AKI was included *a priori* as a co-primary endpoint to evaluate clinical safety alongside biochemical recovery, particularly given the known physiological effects of fluid chloride loads on renal perfusion. Secondary outcomes included hospital length of stay and electrolyte imbalances such as hypokalemia and hyponatremia. Only RCTs were included.

Studies were excluded if they were non-randomized, observational in design, involved adult-only populations, did not directly compare balanced crystalloids with 0.9% saline, or lacked extractable outcome data. In addition, *in vitro* studies, conference abstracts, and animal studies were excluded.

### 2.4 Data extraction

Using a standardized data collection form in Microsoft Excel, two reviewers independently extracted the data. In addition, if any disagreements appeared, they were settled by discussion or consultation with a senior reviewer. The data extraction sheet predefined the extracted variables, which included outcome measures and characteristics at the study and patient levels. First author, country, year of publication, study design, sample size, and trial registration information were among the study characteristics that were extracted. Mean age, sex distribution, diabetes status (newly diagnosed or known), severity of diabetic ketoacidosis, Glasgow Coma Scale (GCS) at admission, and length of disease were among the patient characteristics. Numerous outcomes were retrieved, among them were: time to resolution of DKA, the incidence of new onset acute kidney injury, duration of stay in the hospital, and electrolyte abnormalities, especially hypokalemia and hyponatremia. Where possible, means and standard deviations were derived for continuous results. When appropriate, recognized statistical methods were used to convert data reported as medians and interquartile ranges to means and standard deviations.

### 2.5 Bias and certainty assessments

Using the Cochrane Risk of Bias Tool version 2, two reviewers independently evaluated the risk of bias of included RCTs. The five domains being assessed for bias are: the randomization process, changes from the intended interventions, missing outcome data, outcome measurements, and the selection of reported results. Each domain was assigned an overall risk of bias judgment, and each domain was assessed as “low risk”, “some concerns”, or “high risk of bias”. Conflicts were settled by discussing them or consulting a senior reviewer. The Grading of Recommendations Assessment, Development and Evaluation (GRADE) approach was used to evaluate the certainty of the evidence for each outcome, taking into account publication bias, inconsistency, indirectness, imprecision, and study limitations (9).

### 2.6 Data synthesis

When required, meta-analysis was used to carry out quantitative synthesis. Using extracted means and standard deviations, mean differences (MD) with 95% confidence intervals (CI) were used to pool continuous outcomes, such as the time to resolution of DKA. MD was deemed appropriate because all included studies measured this continuous outcome using an identical unit of measurement (hours), despite variations in the exact clinical parameters used to define DKA resolution. Risk ratios (RR) with 95% confidence intervals were calculated using extracted event counts and total sample sizes for analyzing dichotomous outcomes, including the incidence of new onset AKI and electrolyte abnormalities.

All meta-analyses employed a random effects model to account for between-study variability. Statistical heterogeneity was evaluated using Cochran's *Q* test, and the *I*^2^ statistic was used to quantify it. Rather than relying on a rigid threshold, the presence of heterogeneity was assessed comprehensively by examining clinical differences across trials, the overlap of confidence intervals, and the overall consistency in the direction and size of the treatment effect.

Subgroup analyses were performed based on the different types of balanced crystalloids (like Hartmann's solution, Ringer's lactate, or Plasma-Lyte). Publication bias was not formally assessed, as fewer than 10 trials were available for each outcome (10).

## 3 Results

### 3.1 Study selection

**Figure 1:**
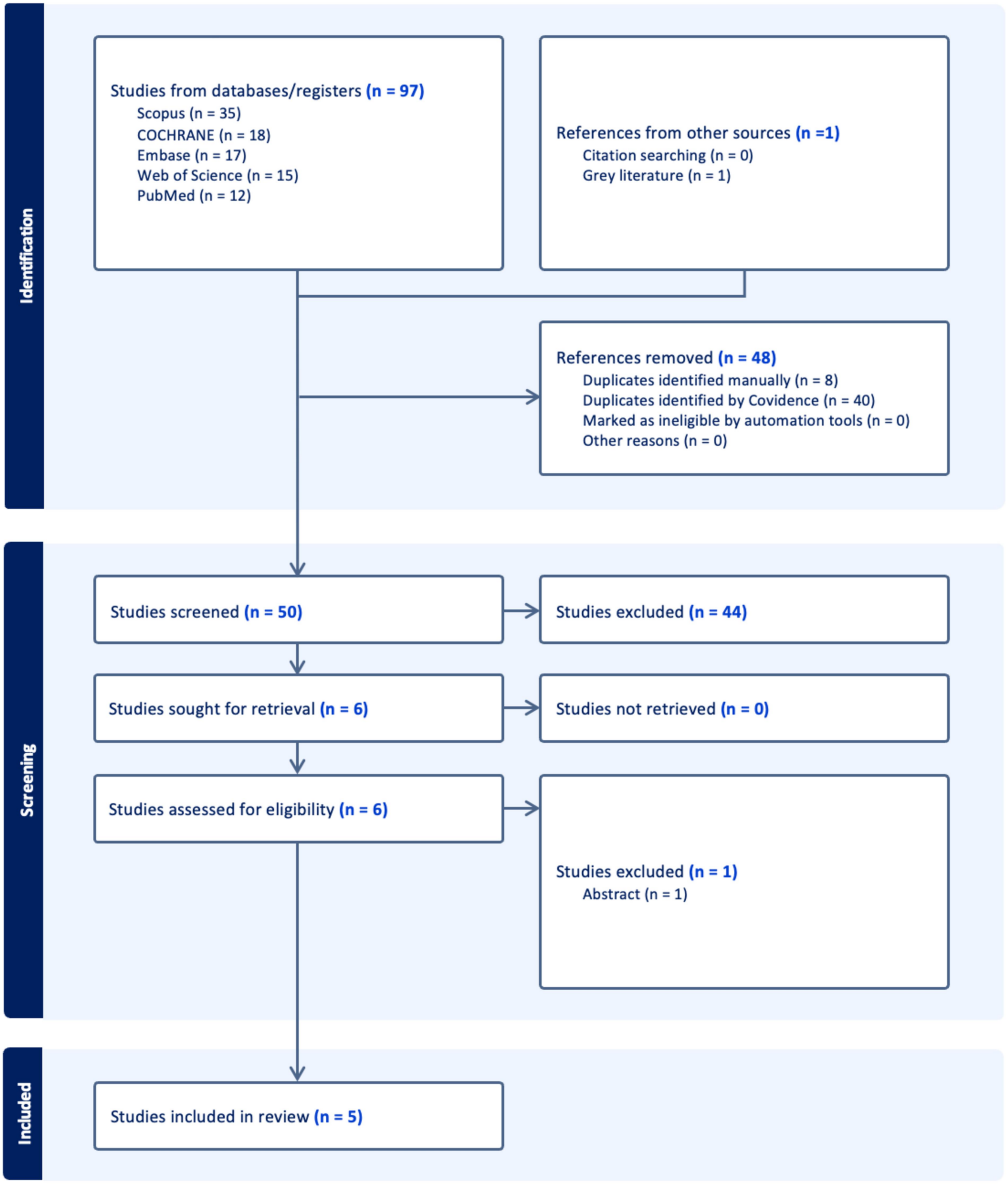
PRISMA 2020 flow diagram of study selection.

The initial literature search across databases and registers identified a total of 98 records, including Scopus (*n* = 35), CENTRAL (*n* = 18), Embase (*n* = 17), Web of Science (*n* = 15), PubMed (*n* = 12), and one additional record from grey literature sources. Following the removal of 48 duplicates (40 identified using Covidence and 8 manually), 50 records underwent title and abstract screening. Of these, 44 records were excluded, and six reports were sought for retrieval. All six full-text articles were successfully retrieved and assessed for eligibility. One study was excluded at the full-text stage due to being an abstract only (11), leaving five RCTs that met the inclusion criteria and were included in the final review (12–16). The complete study selection process is illustrated in the PRISMA flow diagram (Figure 1).

### 3.2 Study characteristics

Our included studies enrolled a total of 320 participants (160 per group) and were conducted across two countries, India and Australia. All studies were single-center trials and employed a double-blinded method except for one, which utilized a triple-blinded design.

Four RCTs used RL as the intervention fluid, while one study used PL. In the treatment of pediatric DKA, all treatments were compared with 0.9% NS. In all research groups, either NS or the designated trial fluid was used for initial fluid resuscitation.

The International Society for Pediatric and Adolescent Diabetes (ISPAD) criteria were used to define DKA in several studies, though the exact versions differed (2014, 2018, and 2022). Similar to this, the criteria of DKA resolution varied significantly throughout studies, but they often included normalization of venous pH (≥7.3), bicarbonate (≥15 mEq/L), and, occasionally, other parameters such as anion gap closure, ketone clearance, and normalization of sensorium.

Four studies used the Kidney Disease Improving Global Outcomes (KDIGO) criteria to measure AKI; one study also used the pediatric RIFLE (p-RIFLE) classification. Time to resolution of acidosis or DKA and incidence of AKI were among the primary outcomes that differed between studies. Secondary outcomes were heterogeneous and included metabolic parameters (anion gap, bicarbonate, chloride), clinical outcomes (PICU and hospital length of stay), and complications such as hypokalemia, cerebral edema, and need for mechanical ventilation. A comprehensive summary of the included studies is presented in Table 1.

**Table 1:** Summary of the included studies.

Study	Country	Study design	Sample size			Initial fluid (resuscitation for both arms)	Intervention details	Control details	Exclusion criteria	Acute kidney injury scale used	Definition of DKA resolution	Primary outcome/s
			Intervention	Control	Total							
Yung et al. (12)	Australia	Double blinded, single-center	38	39	77	Normal saline	Hartmann's solution	Normal saline	Children with moderate–severe DKA (glucose >11 mmol/L, pH <7.3 and/or HCO_3_ < 15 mmol/L with ketosis). Excluded if GCS <11, mechanical ventilation, corrected Na <130 mmol/L, K⁺ > 5.5 mmol/L, or prior enrolment	NR	- Venous HCO3 ≥ 15 mmol/L OR - Venous pH of >7.3	Time taken for resolution of bicarbonate
Williams et al. (13)	India	Double blinded, single-center	34	32	66	Trial fluid (PL or NS)	Plasma-Lyte	Normal saline	Children aged >1 month to <12 years presenting with DKA (ISPAD 2014 criteria). Excluded if symptomatic cerebral edema (GCS <8), chronic kidney or liver disease, or prior fluid/insulin therapy before presentation.	KDIGO AND p-RIFLE AND Urinary NGAL	- Venous pH of >7.3 AND - Bicarbonate > 15 mEq/L AND - Normal sensorium	Incidence of new onset or progressive AKI
Singhal et al. (14)	India	Triple blinded, single-center	25	25	50	Normal saline	Ringer's lactate	Normal saline	Children aged 6 months–18 years with DKA (ISPAD 2022 criteria). Excluded if pre-treated with fluids/insulin, hyponatremia (Na <135 Eq/L), or hyperkalemia (K⁺ > 5 mEq/L).	KDIGO	Venous pH of >7.3	Time taken for resolution of acidosis
Agarwal et al. (15)	India	Double blinded, single-center	33	34	67	Trial fluid (RL or NS)	Ringer's lactate	Normal saline	Children aged 9 months–12 years with DKA (ISPAD 2018 criteria). Excluded if cerebral edema, CKD, liver disease, inborn errors of metabolism, or hyperkalemia (K⁺ > 5.5 mEq/L).	KDIGO	- Venous pH of >7.3 AND - Bicarbonate >15 mEq/L AND - Normal sensorium AND - Blood ketones <2 mmol/L AND - Closure of anion gap (<12 mEq/L)	Time taken for resolution of DKA
Sweety et al. (16)	India	Double blinded, single-center	30	30	60	Trial fluid (RL or NS)	Ringer's lactate	Normal saline	Children aged 5–14 years with DKA (ISPAD 2022 criteria). Excluded if renal, cardiac, or liver disease, cerebral edema, hyponatremia (Na <135), hyperkalemia (K⁺ > 5.5), contraindications to fluids, or prior treatment.	KDIGO	Venous pH of >7.3	Time taken for resolution of acidosis

AKI, acute kidney injury; KDIGO, Kidney Disease: Improving Global Outcomes; p-RIFLE, Pediatric Risk, Injury, Failure, Loss, End-stage kidney disease; NGAL, neutrophil gelatinase-associated lipocalin; NR, not reported.

### 3.3 Patients baseline demographics

Baseline demographic characteristics were broadly comparable between intervention and control groups. Mean age of arms ranged between 5.67 and 13.1. The percentage of male participants varied from 23.3% to 62%, whereas the percentage of female participants varied from 38% to 76.7%, indicating some variation in the gender distribution among the research participants. Not all studies reported the weight and height of patients. However, when reported, no major imbalance between groups was noticed. Mean weight of patients ranged from 16.3 to 42.7 kg, while mean height ranged from 106.33 to 131.2 cm. Although the number of known cases of diabetes was reported inconsistently, it varied between groups. The Glasgow Coma Scale (GCS), which measures neurological state upon presentation, showed comparable results across studies, with mean scores often suggesting mild to severe impairment.

Despite differences in reporting and categorization methods, disease severity at presentation was generally in the moderate to severe range in all trials. The mean duration of diabetes ranged from 2.27 to 5 years in studies reporting the condition's duration, indicating a combination of newly diagnosed and previously identified cases.

All of the trials' baseline laboratory results showed moderate to severe DKA. The mean pH values ranged from 7.01 to 7.15, suggesting severe acidity, whereas the mean blood glucose levels varied from 392.4 to 513.7 mg/dL. The mean potassium levels varied between 3.7 and 4.7 mmol/L, while blood ketone levels were increased when reported. Additional biochemical markers, including anion gap, lactate, and osmolality, were broadly comparable between groups, with no clinically meaningful baseline imbalances observed (Table 2).

**Table 2:** Baseline characteristics of the included studies.

Study	Arm	Age (years), Mean/SD	Gender		Height (cm), Mean/SD	Weight (kg), Mean/SD	Known Case of Diabetes, (n/%)	GCS at admission, Mean/SD	Severity of DKA at presentation (n/%)			Duration of Disease (years), Mean/SD	Laboratory Parameters									
			Boys (n/%)	Girls (n/%)					Severe	Moderate	Mild		HbA1c (%), Mean/SD	Blood Glucose (mg/dL), Mean/SD	Blood pH, Mean/SD	Blood Ketone (mmol/L), Mean/SD	Potassium (mmol/L), Mean/SD	Lactate (mmol/L), Mean/SD	Anion Gap (mmol/L), Mean/SD	Osmolality (mmol/kg), Mean/SD	pCO2 (mmHg), Mean/SD	pO2 (mmHg), Mean/SD
Yung et al. (12)	Intervention	13.10 ± 2.85	23 (61)	15 (39)	NR	42.70 ± 17.10	19 (50)	NR	13 (34)	NR	NR	NR	NR	392.40 ± 113.40	7.11 ± 0.12	5.37 ± 0.69	4.70 ± 1.00	NR	26.10 ± 4.10	320.00 ± 15.00	NR	NR
	Control	11.97 ± 5.00	24 (62)	15 (38)	NR	39.70 ± 19.60	19 (49)	NR	16 (41)	NR	NR	NR	NR	401.40 ± 104.40	7.08 ± 0.19	5.23 ± 0.77	4.30 ± 0.70	NR	26.30 ± 4.50	317.00 ± 14.00	NR	NR
Williams et al. (13)	Intervention	7.80 ± 5.88	18 (53)	16 (47)	NR	NR	NR	13.33 ± 3.10	20 (59)	11 (32)	3 (9)	2.27 ± 2.51	12.48 ± 2.13	513.67 ± 141.63	7.01 ± 0.20	5.70 ± 1.24	4.10 ± 1.08	1.93 ± 0.93	19.60 ± 5.26	300.33 ± 11.61	21.47 ± 8.98	NR
	Control	6.53 ± 5.59	15 (47)	17 (53)	NR	NR	NR	12.67 ± 3.88	20 (63)	11 (34)	1 (3)	1.49 ± 1.61	11.58 ± 2.69	424.67 ± 85.36	7.01 ± 0.19	5.40 ± 1.32	3.80 ± 0.93	1.90 ± 0.70	19.00 ± 5.04	299.00 ± 11.64	19.90 ± 7.37	NR
Singhal et al. (14)	Intervention	9.67 ± 5.50	12 (48)	13 (52)	NR	21.33 ± 10.22	20 (80)	15 ± 0	8 (32)	9 (36)	8 (32)	NR	10.07 ± 2.12	406.67 ± 144.65	7.15 ± 0.10	4.30 ± 1.34	NR	1.77 ± 0.86	23.33 ± 5.50	297.00 ± 10.22	25.33 ± 8.65	47.00 ± 21.23
	Control	7.33 ± 7.08	7 (28)	18 (72)	NR	18.33 ± 10.22	20 (80)	14.67 ± 0.79	12 (48)	6 (24)	7 (28)	NR	10.23 ± 3.38	458.67 ± 73.90	7.10 ± 0.22	4.60 ± 1.42	NR	1.77 ± 0.94	22.00 ± 7.08	302.00 ± 9.43	21.67 ± 10.22	54.00 ± 13.36
Agarwal et al. (15)	Intervention	5.67 ± 3.87	10 (30)	23 (70)	106.33 ± 30.22	16.33 ± 6.59	NR	12.67 ± 3.87	23 (70)	5 (15)	5 (15)	3.00 ± 2.96	12.33 ± 2.25	458.67 ± 133.29	7.01 ± 0.15	5.13 ± 0.70	4.27 ± 0.62	2.03 ± 0.93	15.73 ± 2.94	292.03 ± 7.83	19.17 ± 5.81	NR
	Control	6.67 ± 3.87	13 (38)	21 (62)	115.00 ± 23.22	16.80 ± 5.57	NR	13.67 ± 3.10	20 (59)	6 (17)	8 (24)	2.58 ± 2.78	12.47 ± 2.01	483.33 ± 111.45	7.04 ± 0.19	5.37 ± 1.63	3.93 ± 0.93	1.97 ± 1.01	17.35 ± 3.83	294.27 ± 10.22	19.57 ± 4.88	NR
Sweety et al. (16)	Intervention	10.77 ± 1.36	7 (23)	23 (77)	131.20 ± 24.10	19.20 ± 6.50	10 (33)	13.00 ± 2.00	21 (70)	7 (23)	2 (7)	5.00 ± 2.00	10.20 ± 2.49	454.67 ± 143.23	7.03 ± 0.22	NR	3.83 ± 0.62	2.00 ± 1.56	16.67 ± 4.67	299.00 ± 14.79	19.33 ± 6.23	NR
	Control	10.63 ± 1.50	9 (30)	21 (70)	130.90 ± 24.30	19.50 ± 6.40	11 (37)	13.00 ± 2.00	20 (67)	8 (26)	2 (7)	5.00 ± 2.00	9.90 ± 2.26	448.00 ± 146.34	7.04 ± 0.23	NR	3.73 ± 0.54	2.00 ± 1.56	16.67 ± 3.89	300.33 ± 13.23	19.00 ± 4.67	NR

GCS, Glasgow Coma Scale; DKA, diabetic ketoacidosis; HbA1c, glycated hemoglobin; SD, standard deviation; NR, not reported; pCO_2_, partial pressure of carbon dioxide; pO_2_, partial pressure of oxygen.

### 3.4 Primary outcomes

**Figure 2:**
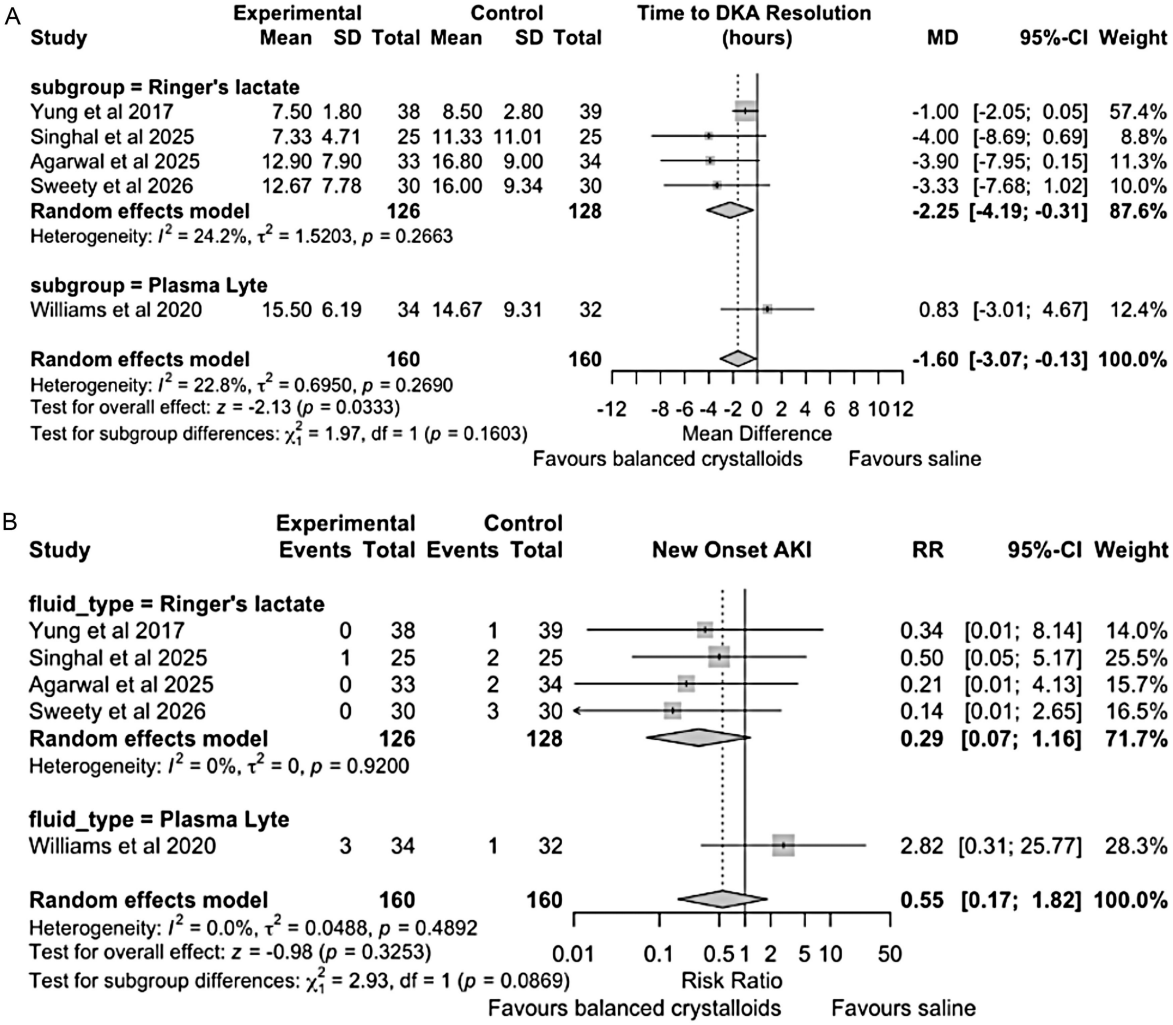
Forest plots of primary outcomes comparing balanced crystalloids with normal saline in pediatric diabetic ketoacidosis. **(A)** Time to DKA resolution (hours), presented as mean difference (MD). **(B)** New-onset acute kidney injury (AKI), presented as risk ratio (RR). Subgroup analyses were performed based on fluid type [Ringer's lactate (RL) and Plasma-Lyte (PL)]. Squares represent individual study estimates, with sizes proportional to study weight, and horizontal lines indicate 95% confidence intervals (CI). Diamonds represent pooled estimates using a random-effects model. Values <0 (for MD) and <1 (for RR) favor balanced crystalloids.

For time to DKA resolution, the pooled analysis included 320 patients from five RCTs (12–16). Balanced crystalloids were associated with a statistically significant reduction in time to DKA resolution compared with normal saline (MD: −1.60 h, 95% CI: −3.07 to −0.13, *p* = 0.033), with low heterogeneity (*I*^2^ = 22.8%) (Figure 2A). Subgroup analysis showed a reduction in time to DKA resolution with Ringer's lactate (MD: −2.25 h, 95% CI: −4.19 to −0.31), whereas no significant difference was observed in the Plasma-Lyte subgroup (MD: 0.83 h, 95% CI: −3.01 to 4.67). There was no significant difference between subgroups (*p* for interaction = 0.16). Definitions of DKA resolution varied across studies, with some using resolution of acidosis (pH > 7.3) and others employing composite criteria (Table 1).

For new-onset AKI, all five RCTs contributed to the analysis. The pooled estimate for new-onset AKI favored balanced crystalloids but was accompanied by substantial statistical uncertainty (RR: 0.55, 95% CI: 0.17–1.82, *p* = 0.325; *I*^2^ = 0%), making the data compatible with both clinical benefit and harm due to the small sample size and low event rates (Figure 2B). Subgroup analysis showed a lower risk of AKI with Ringer's lactate (RR: 0.29, 95% CI: 0.07–1.16), whereas the Plasma-Lyte subgroup showed a higher, but non-significant, risk (RR: 2.82, 95% CI: 0.31–25.77). There was no significant difference between subgroups (*p* for interaction = 0.087). Four RCTs defined AKI using the KDIGO criteria, while one study (Yung et al.) did not specify a standardized definition (Table 1).

### 3.5 Secondary outcomes

#### Length of hospital stay

The pooled analysis for length of hospital stay included 176 patients from three RCTs. Balanced crystalloids were not associated with a statistically significant reduction in hospital stay compared with normal saline (MD: −0.52 days, 95% CI: −1.16 to 0.11, *p* = 0.107), with no observed heterogeneity (*I*^2^ = 0%) (Supplementary Figure S1A). Subgroup analysis showed no significant difference in either the RL subgroup (MD: −0.67 days, 95% CI: −1.40 to 0.06) or the PL subgroup (MD: −0.08 days, 95% CI: −1.36 to 1.20), with no significant difference between subgroups (*p* for interaction = 0.43).

#### Length of PICU stay

Data from three RCTs (*n* = 193) were included. Balanced crystalloids were not associated with a statistically significant difference in PICU stay compared with normal saline (MD: −0.69 h, 95% CI: −13.49 to 12.12, *p* = 0.916), with substantial heterogeneity (*I*^2^ = 80.5%) (Supplementary Figure S1B). Subgroup analysis showed no significant difference in the RL subgroup (MD: −4.24 h, 95% CI: −8.68 to 0.20), whereas the PL subgroup showed a significantly longer PICU stay (MD: 10.33 h, 95% CI: 1.68–18.98). A significant difference between subgroups was observed (*p* for interaction = 0.003).

#### Required inotropes

The pooled analysis for inotrope requirement included 187 patients from three RCTs. Balanced crystalloids were not associated with a statistically significant difference in the need for inotropes compared with normal saline (RR: 1.41, 95% CI: 0.28–7.07, *p* = 0.680), with no observed heterogeneity (*I*^2^ = 0%) (Supplementary Figure S2A).

#### Required ventilation

Analysis of the requirement for mechanical ventilation included 176 patients from three RCTs. Balanced crystalloids were not associated with a statistically significant difference in ventilation requirement compared with normal saline (RR: 1.29, 95% CI: 0.29–5.71, *p* = 0.739), with no observed heterogeneity (*I*^2^ = 0%) (Supplementary Figure S2B). Subgroup analysis showed no significant difference in either the RL subgroup (RR: 1.00, 95% CI: 0.15–6.85) or the PL subgroup (RR: 1.88, 95% CI: 0.18–19.77), with no significant difference between subgroups (*p* for interaction = 0.68).

#### Cerebral oedema

The pooled analysis for cerebral edema included 176 patients from three RCTs. Balanced crystalloids were not associated with a statistically significant difference in the risk of cerebral edema compared with normal saline (RR: 0.55, 95% CI: 0.09–3.29, *p* = 0.513), with no observed heterogeneity (*I*^2^ = 0%) (Supplementary Figure S2C). Subgroup analysis showed no significant difference in either the RL subgroup (RR: 0.25, 95% CI: 0.03–2.23) or the PL subgroup (RR: 2.83, 95% CI: 0.12–66.92), with no significant difference between subgroups (*p* for interaction = 0.22).

#### Electrolyte disturbances

Balanced crystalloids were associated with a statistically significant reduction in hypokalemia (RR: 0.66, 95% CI: 0.46–0.93, *p* = 0.019), while no significant differences were observed for hyponatremia (RR: 1.01, 95% CI: 0.52–1.95, *p* = 0.977) or hypoglycemia (RR: 0.93, 95% CI: 0.43–2.01, *p* = 0.862). A significant reduction in hyperchloremia was observed with balanced crystalloids (RR: 0.40, 95% CI: 0.21–0.78, *p* = 0.007). No heterogeneity was observed across these analyses (*I*^2^ = 0%) (Supplementary Figures S3A–D).

### 3.6 Quality assessment and certainty of evidence

**Figure 3:**
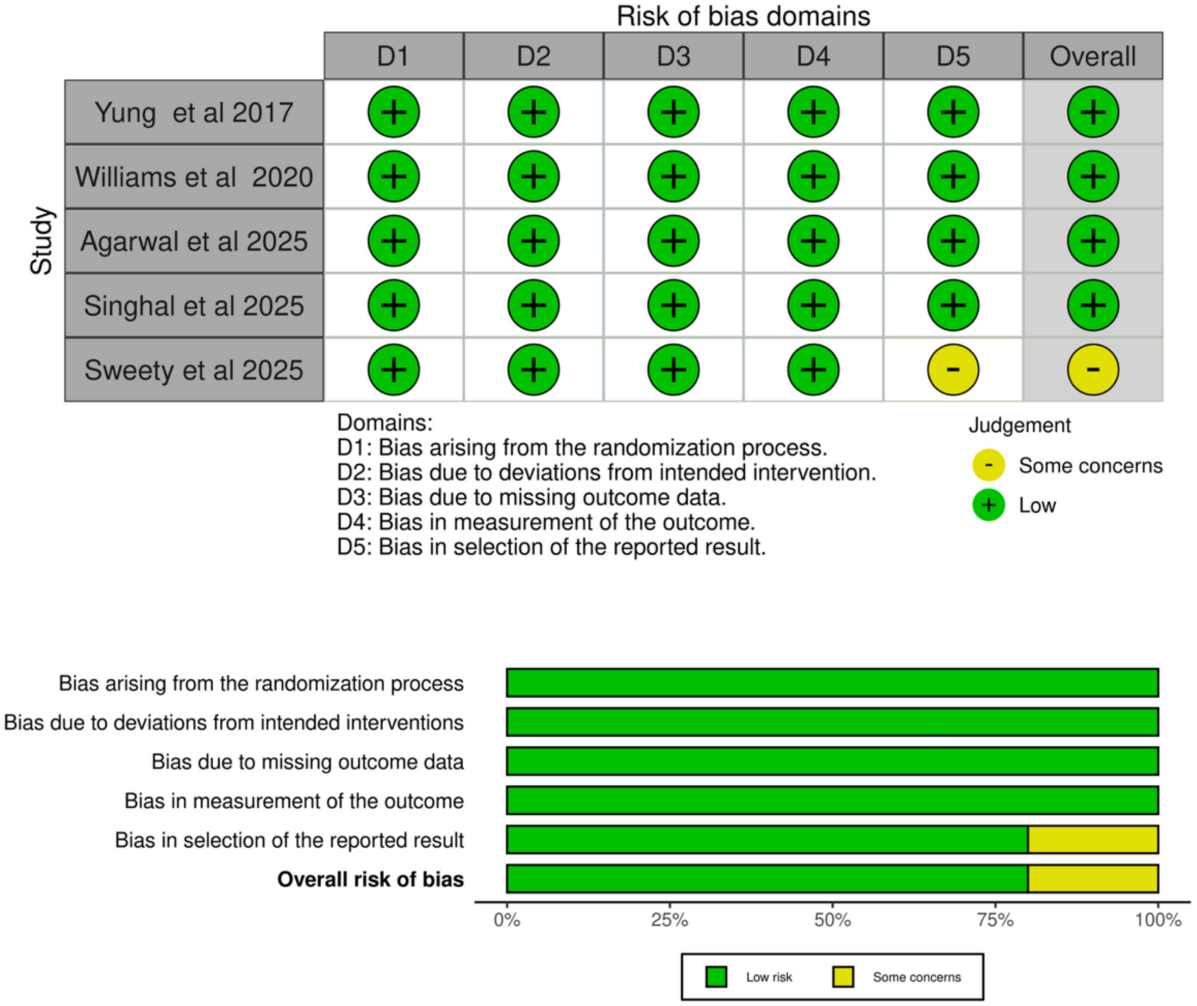
Risk of bias assessment of included studies using the Cochrane RoB 2 tool. The upper panel shows the risk of bias summary for individual studies across five domains: bias arising from the randomization process (D1), deviations from intended interventions (D2), missing outcome data (D3), measurement of the outcome (D4), and selection of the reported result (D5), along with overall risk of bias. The lower panel shows the risk of bias graph, illustrating the proportion of studies classified as low risk or with some concerns across each domain and overall. Green indicates low risk of bias, and yellow indicates some concerns.

Overall, the risk of bias across included studies was low (Figure 3). All trials were judged to have low risk of bias in the domains of randomization, deviations from intended interventions, missing outcome data, and outcome measurement. One study (Sweety et al.) had some concerns in the domain of selection of the reported result, resulting in an overall judgment of some concerns. The remaining studies were assessed as having a low overall risk of bias.

The certainty of evidence for primary outcomes was rated as low (Table 3). Evidence for time to DKA resolution was rated as low certainty, downgraded for both imprecision and indirectness owing to variability in resolution criteria across the included trials, while evidence for new-onset AKI was rated as low certainty due to serious imprecision. Among secondary outcomes (Supplementary Table S3), the certainty of evidence ranged from moderate to very low. Hypokalemia and hospital length of stay were rated as moderate certainty, whereas most other outcomes, including hyponatremia, hyperchloremia, and hypoglycemia, were rated as low certainty due to imprecision. Outcomes with very few events, including inotrope requirement, mechanical ventilation, and cerebral edema, were rated as very low certainty. The certainty of evidence for PICU length of stay was also very low due to both inconsistency and imprecision.

**Table 3:** GRADE assessment of primary outcomes.

Outcome	Relative/Absolute Effect	No. of Participants (Studies)	Certainty of the Evidence (GRADE)	Comments
Time to DKA resolution (hours)	Mean difference −1.60 h (95% CI −3.07 to −0.13)	320 (5 RCTs)	LOW	Downgraded for imprecision (upper CI bound approaches the null; modest absolute effect) and indirectness (variability in DKA resolution criteria across trials, ranging from venous pH > 7.3 alone to multi-component composite definitions). Low heterogeneity (*I*^2^ = 22.8%); favours balanced crystalloids.
			⬤⬤◯◯	
New-onset acute kidney injury (AKI)	Risk ratio 0.55 (95% CI 0.17 to 1.82)	320 (5 RCTs)	LOW	Downgraded for serious imprecision (few events, wide CI crossing no effect). No heterogeneity (*I*^2^ = 0%).
			⬤⬤◯◯	

DKA, diabetic ketoacidosis; CI, confidence interval; RCTs, randomized controlled trials; GRADE, Grading of Recommendations Assessment, Development and Evaluation; I², I-squared statistic.

## 4 Discussion

### 4.1 Summary of findings

This systematic review and meta-analysis synthesized available data from five RCTs comparing balanced crystalloids with normal saline in the management of pediatric DKA. A small but statistically significant decrease in the time to DKA resolution was linked to balanced crystalloids, such as RL and PL. However, due to a small evidence base and low event rates, current data are insufficient to determine whether fluid choice truly impacts new-onset AKI. Subgroup analysis revealed that the PL subgroup had a longer PICU stay, but given the small sample size and significant variability, this result should be taken cautiously. There was a trend toward shorter hospital stays with balanced crystalloids, although it was not statistically significant. Furthermore, balanced crystalloids were linked to a lower incidence of electrolyte imbalances, especially hyperchloremia and hypokalemia; hypoglycemia and hyponatremia did not vary significantly. Severe problems, such as the need for inotropes and ventilatory support, did not vary between groups significantly. However, due to the small number of occurrences, the evidence power is insufficient to confirm safety, resulting in a very low certainty of evidence for these complications.

### 4.2 Interpretation of findings and comparison with existing literature

Current guidelines emphasize that fluid resuscitation is a cornerstone of acute DKA management, as it restores circulating volume, facilitates clearance of ketones, and corrects electrolyte imbalances (17, 18). Despite its high chloremic content, 0.9% NS remains the fluid of choice for managing DKA in adults and pediatric populations (17, 19). However, a recent meta-analysis has found that balanced crystalloids like RL and PL might offer an advantage when it comes to biochemical profile compared to NS in adult populations (20). In this context, our findings provide pediatric-specific evidence that partially aligns with emerging data from adult populations.

Our study found that balanced crystalloids modestly reduce time to DKA resolution, which may be explained by their lower chloride content, which in turn is less likely to induce hyperchloremic metabolic acidosis (HMA). The use of large amounts of fluids rich in chlorine, like NS, has been associated with HMA, which may contribute to a slower correction of acid–base status (2). Furthermore, hyperchloremia may result in a persistent base deficit despite resolution of ketosis, potentially delaying the apparent resolution of DKA when bicarbonate-based criteria are used (2). Evidence showing that biochemical indicators of DKA resolution, such as venous pH, anion gap, and bicarbonate, normalize at various speeds lends credence to this. Therefore, the identification of real metabolic recovery may be delayed if bicarbonate-based criteria are relied upon (21). The observed decrease in hyperchloremia and hypokalemia in our results provides more evidence for this.

Balanced crystalloids did not enhance clinically significant results despite these physiological benefits. Due to the complex nature of kidney damage in DKA and the small number of occurrences, there may not have been a meaningful decrease in the incidence of new-onset AKI. According to available data, renal perfusion, underlying metabolic disorders, and the degree of dehydration all have an impact on kidney damage in DKA, rather than just fluid composition (22). This is especially important since elevated chloride load has been linked to renal vasoconstriction through the tubuloglomerular feedback pathway, which might lower the glomerular filtration rate (23). This impact might not be enough, though, to result in detectable variations in clinical outcomes.

The biochemical advantage did not result in significant clinical advantages, such as decreased hospital/PICU stays or complication rates, even though balanced crystalloids were more successful than NS in balancing electrolytes and shortening the time to DKA resolution. This might be explained by the fact that recovery from DKA is complex and depends on insulin delivery, electrolyte replenishment, fluid treatment, and general clinical care (16). While variations in severe complications, including cerebral edema, the necessity for mechanical ventilation, and the need for inotropes, were not observed, the very low certainty of evidence for these outcomes limits our ability to draw definitive conclusions. However, current knowledge of the pathophysiology of DKA is consistent with the lack of variation in consequences, including cerebral edema, the necessity for mechanical ventilation, and the need for inotropes. According to available data, cerebral hypoperfusion, neuroinflammation, and the degree of metabolic abnormalities at presentation, rather than fluid type or administration rate, are the main causes of brain damage in DKA (24). This may explain why variations in fluid composition did not translate into differences in neurological or critical care outcomes in our analysis.

Cost is a significant factor to take into account while choosing a fluid. Since 0.9% normal saline is inexpensive and widely available in many healthcare settings, it is recommended as the first-line fluid in current DKA therapy recommendations (25, 26). In the context of our findings, balanced crystalloids did not demonstrate clear superiority in clinically important outcomes, and therefore, the cost-effectiveness of NS may remain an important factor in deciding on the choice of fluid administered, especially in resource-limited settings.

Taken together, these findings highlight the distinction between improvements in biochemical parameters and clinically meaningful outcomes. Overall, our findings support previous research that suggests balanced crystalloids may enhance biochemical outcomes without necessarily converting those improvements into benefits that are clinically significant. While our findings support the physiological rationale for the use of balanced fluids, they do not necessarily provide sufficient evidence to warrant a change in current guideline-recommended practice.

### 4.3 Strengths and limitations

To our knowledge, this is the first systematic review and meta-analysis to comprehensively compare balanced crystalloids with normal saline in pediatric DKA. This review's limitation to randomized controlled trials that directly evaluated both therapies is a major strength that improves the internal validity of our conclusions. Additional information on the individual balanced crystalloids, particularly RL and PL, was evaluated through fluid-type subgroup analyses. However, a major limitation of these evaluations is the severe data imbalance, with only one trial investigating PL compared to four for RL. These subgroup analyses are therefore inherently underpowered and must be interpreted as exploratory and hypothesis-generating rather than definitive evidence of differences. Furthermore, the GRADE framework was used to carefully evaluate the certainty of the evidence, resulting in a clear assessment of the strength and limits of our results, and the overall risk of bias among included studies was minimal.

This review is limited in a number of ways. First, there were only five RCTs in the total sample size, which was rather small. This reduced the studies' statistical power, especially for uncommon outcomes including cerebral edema, the need for inotropes, and mechanical ventilation. Second, a small number of studies reported a lot of outcomes, which led to large confidence intervals and increased the pooled estimates' imprecision. Thirdly, different researchers used different definitions of DKA resolution, with most relying heavily on pH and bicarbonate normalization. As normal saline inherently delays the normalization of these specific parameters due to hyperchloremic metabolic acidosis, this acts as a systematic confounding factor that may artificially bias the “time to resolution” outcome in favor of balanced crystalloids. Additionally, variations in clinical procedures, patient groups, and fluid management strategies may be reflected in the variability found in some outcomes, such as PICU duration of stay. Although only randomized controlled trials were included and the overall risk of bias was low, the certainty of evidence for several outcomes remained low to very low, primarily due to imprecision and low event rates. Finally, because our review includes fewer than ten trials, we could not formally test for publication bias.

## 5 Conclusion

Compared to 0.9% normal saline, balanced crystalloids were linked to a somewhat shorter time to DKA resolution and a decreased risk of specific electrolyte imbalances, especially hyperchloremia and hypokalemia, in this comprehensive review and meta-analysis of randomized controlled trials. However, the current evidence base is too small to definitively conclude whether these biochemical benefits translate to improvements in clinically meaningful outcomes. Current findings do not support a change in guideline-recommended practice favoring normal saline as the first fluid in pediatric DKA due to the moderate to low certainty of the available evidence and the lack of a clear therapeutic advantage. Although balanced crystalloids could have physiological benefits, it is unclear how they might enhance clinically significant results. Future large, multicenter randomized trials are needed to better clarify the clinical role of balanced crystalloids in pediatric DKA.

## Data Availability

The original contributions presented in the study are included in the article/Supplementary Material, further inquiries can be directed to the corresponding author.
